# Serum cystatin C and inflammatory factors related to COVID-19 consequences

**DOI:** 10.1186/s12879-023-08258-0

**Published:** 2023-05-22

**Authors:** Azadeh Mottaghi, Farzaneh Alipour, Nazanin Alibeik, Ali Kabir, Shokoufeh Savaj, Ramin Bozorgmehr, Mehdi Nikkhah, Neda Rahimian

**Affiliations:** 1 Research Center for Prevention of Cardiovascular diseases, Institute of Endocrinology Metabolism, Iran University of Medical Sciences, Tehran, Iran; 2 Department of Internal Medicine, Firoozgar Hospital, School of Medicine, Iran University of Medical Sciences, Tehran, Iran; 3 Minimally Invasive Surgery Research Center, Iran University of Medical Sciences, Tehran, Iran; 4 Department of Nephrology, Firoozgar Hospital, School of Medicine, Iran University of Medical Sciences, Tehran, Iran; 5 Department of Surgery, School of Medicine, Shahid Madani Hospital, Alborz University of Medical Sciences, Karaj, Iran; 6 Gastrointestinal and Liver Diseases Research Center, Iran University of Medical Sciences, Tehran, Iran

**Keywords:** Covid-19, Extra-polmunary, Acute kidnet injury, Inflammatory biomarkers

## Abstract

**Background:**

Besides impaired respiratory function and immune system, COVID-19 can affect renal function from elevated blood urea nitrogen (BUN) or serum creatinine (sCr) levels to acute kidney injury (AKI) and renal failure. This study aims to investigate the relationship between Cystatin C and other inflammatory factors with the consequences of COVID-19.

**Methods:**

A total of 125 patients with confirmed Covid-19 pneumonia were recruited in this cross-sectional study from March 2021 to May 2022 at Firoozgar educational hospital in Tehran, Iran. Lymphopenia was an absolute lymphocyte count of less than 1.5 × 109/L. AKI was identified as elevated serum Cr concentration or reduced urine output. Pulmonary consequences were evaluated. Mortality was recorded in the hospital one and three months after discharge. The effect of baseline biochemical and inflammatory factors on odds of death was examined. SPSS, version 26, was used for all analyses. P-vale less than 0.05 was considered significant.

**Results:**

The highest amount of co-morbidities was attributed to COPD (31%; n = 39), dyslipidemia and hypertension (27%; n = 34 for each) and diabetes (25%; n = 31). The mean baseline cystatin C level was 1.42 ± 0.93 mg/L, baseline creatinine was 1.38 ± 0.86 mg/L, and baseline NLR was 6.17 ± 4.50. Baseline cystatin C level had a direct and highly significant linear relationship with baseline creatinine level of patients (*P < 0.001*; r: 0.926). ). The average score of the severity of lung involvement was 31.42 ± 10.80. There is a direct and highly significant linear relationship between baseline cystatin C level and lung involvement severity score (r = 0.890, *P < 0.001*). Cystatin C has a higher diagnostic power in predicting the severity of lung involvement (B = 3.88 ± 1.74, p = 0.026). The mean baseline cystatin C level in patients with AKI was 2.41 ± 1.43 mg/L and significantly higher than patients without AKI (P > 0.001). 34.4% (n = 43) of patients expired in the hospital, and the mean baseline cystatin C level of this group of patients was 1.58 ± 0.90 mg/L which was significantly higher than other patients (1.35 ± 0.94 mg/L, *P = 0.002*).

**Conclusion:**

cystatin C and other inflammatory factors such as ferritin, LDH and CRP can help the physician predict the consequences of COVID-19. Timely diagnosis of these factors can help reduce the complications of COVID-19 and better treat this disease. More studies on the consequences of COVID-19 and knowing the related factors will help treat the disease as well as possible.

## Introduction

In March 2020, the World Health Organization (WHO) confirmed and announced the pandemic and globalization of COVID-19, involving more than 195 countries worldwide. This disease is very contagious, and human-to-human transmission is unfortunately very high. To control this problem, different countries formulated and implemented different quarantine, military, and city protocols to control the movement of citizens.

COVID-19 has manifested in a wide spectrum, from asymptomatic infection to critical pneumonia with acute respiratory distress syndrome (ARDS) [1]. For this reason, multiple organ dysfunctions and mortality are very high, especially in adults with comorbidities [2, 3]. Terrible cytokine storm can make the situation of COVID-19 dangerous due to overactive inflammation and immune response [4]. Besides impaired respiratory function and immune system, COVID-19 can affect renal function from elevated blood urea nitrogen (BUN) or serum creatinine (sCr) levels to acute kidney injury (AKI) and renal failure [5]. Most nucleated cells secret Cystatin C, a cysteine protease inhibitor to blood circulation, and evidence shows that Cystatin C directly interacts with human coronaviruses [6]. Considering that it is not affected by ingestion of meat and no tubular secretion of cystatin, it is a more sensitive biomarker for early kidney impairment than conventional indicators such as BUN and sCr [7].

Ferritin, C-reactive protein (CRP), lactate dehydrogenase (LDH), sCr, and neutrophil to lymphocyte ratio (NLR) were other inflammatory factors investigated. Retrospective analyses found that CRP, ferritin, and LDH were higher in patients who died than in survivors [8, 9].

Through direct immune-suppressive and pro-inflammatory effects, Ferritin has a key role in the dysregulation of the immune system, especially under extreme hyperferritinemia [10]. Results of some studies showed that serum ferritin levels were closely related to the severity of COVID-19 [11–14].

NLR is one routinely available marker of the systemic inflammatory response, which is obtained by dividing the absolute count of neutrophils into the absolute count of lymphocytes in whole blood. Increasing NLR is a mortality risk factor not only in infectious diseases like COVID-19 but also in cancer, acute coronary syndrome, intracerebral hemorrhage, polymyositis, and dermatomyositis [15–18]. There is evidence that patients with severe COVID-19 have higher NLR [19], and more studies are needed to reveal the role of these inflammatory factors in predicting the events caused by COVID-19.

This study aims to investigate the relationship between Cystatin C and other inflammatory factors with the consequences of COVID-19.

## Materials and methods

The present study was done as cross-sectional in a single center from March 2021 to May 2022 at Firoozgar educational hospital in Tehran, Iran. A total of 125 patients 16 to 80 years old with moderate to severe confirmed COVID-19, according to WHO interim guidance [20], were recruited in this study.

This study was approved by the Ethics Committee of the Iran University of Medical IR.IUMS.FMD.REC.1401.068, all patients or first-degree family members signed written informed consent. Smoking status, body mass index (BMI), marital status, and past medical histories like diabetes, hypertension, and dyslipidemia were recorded. Ferritin, C-reactive protein (CRP), Lactate dehydrogenase (LDH), creatinine, and neutrophil to lymphocyte ratio (NLR) were defined.

Lymphopenia was an absolute lymphocyte count of less than 1.5 × 109/L. AKI was identified as elevated serum Cr concentration or reduced urine output. AKI currently defines by a rise from baseline of at least 0.3 mg/dl within 48 h or at least 50% higher than baseline within 1 week, or a reduction in urine output to < 0.5 ml/kg per hour for longer than 6 h [21].

Pulmonary consequences were assessed by rapid shallow breathing index (RSBI), Pressure of Arterial Oxygen to Fractional Inspired Oxygen Concentration (Pao_2_/Fio_2_ ratio), Fractional Inspired Oxygen Concentration (FIO_2_), Oxygen saturation, and respiratory rate. AKI was identified as a renal consequence of COVID-19.

Mortality was recorded in the hospital one and three months after discharge. The effect of baseline biochemical and inflammatory factors on odds of death was examined.

### Statistical analysis

Continuous measurements were reported as mean ± Standard deviation (SD) for normally distributed data, and categorical variables were described as several patients (percent). Continuous variables were compared using an independent sample t-test or Mann-Whitney test based on normality, and proportions for categorical variables were compared using the χ^2^ test. In contrast, the Fisher exact test was conducted when the data were limited. Spearman test was used for analyzing bivariate correlation. Logistic regression was used to evaluate the incidence of AKI and in-hospital death. A generalized linear model was used to show the simultaneous effect of baseline cystatin C and creatinine values on the severity of lung involvement. Our analysis used a 95% confidence interval to express the association’s severity. SPSS, version 26, was used for all analyses. P-vale less than 0.05 was considered significant.

## Results

The present study shows clinical information of 125 hospitalized COVID-19 patients. The mean age of patients was 51.93 ± 19.5 years old. The most of patients were male (57%; n = 71) and married (57%; n = 71). The mean BMI was 26.34 ± 5.4. The highest amount of co-morbidities was attributed to COPD (31%; n = 39), dyslipidemia and hypertension (27%; n = 34 for each) and diabetes (25%; n = 31). Table 1.

**Table 1:** Basic characteristics of Covid-19 patients who are ICU admitted at Firoozgar Hospital

Age; year	51.93 ± 19.50
Sex; N (%)	
Male	71(57)
Female	54(43)
**Marital status; N (%)**	
Single	28(22)
Married	71(57)
Widow	26(21)
**Positive report for smoking; n (%)**	43(34)
**BMI; kg/m2, mean ± SD**	26.3 ± 5.43 (25.38, 27.30)
**Positive report for comorbidities; n (%)**	
Diabetic	31(25)
Hypertension	34(27)
Dyslipidemia	34(27)
Cardiovascular disease	29(23)
Asthma	23(18)
Chronic obstructive pulmonary disease (COPD)	39(31)
Chronic kidney disease (CKD)	18(14)
Liver diseases	13(10)

**Kg: kilogram/meter/2**

**BMI: body mass index**

The mean baseline cystatin C level was 1.42 ± 0.93 mg/L, baseline creatinine was 1.38 ± 0.86 mg/L, and baseline NLR was 6.17 ± 4.50. The level of other biochemical and respiratory indicators is illustrated in Table 2.

**Table 2:** Biochemical and respiratory indexes of Covid-19 patients

Indexes	Basic value		Follow up value	
	N	Values mean ± SD		N
**Temperature, °C**	49	25.62 ± 7.27	49	25.62 ± 7.27
***Respiratory Indexes***				
**Breath rate**	125	25.62 ± 7.27	49	13.77 ± 2.32
**RSBI**	125	95.16 ± 23.81	49	61.87 ± 16.87
**Pao2/Fio2 ratio**	125	216.38 ± 105.03	49	377.55 ± 40.59
**FIO2, %**	125	80.53 ± 21.60	49	31.46 ± 8.18
**Oxygen saturation**	125	82.97 ± 7.39	49	94.97 ± 3.20
***Biochemical indexes***				
**Cystatin C**	125	1.42 ± 0.92	49	-------------
**Creatinine**	125	1.38 ± 0.85	49	-------------
**NLR**	125	6.17 ± 4.50	49	2.80 ± 1.257
**Ferritin**	125	625.77 ± 410.51	49	201.32 ± 163.73
**CRP**	125	89.96 ± 57.27	49	17.38 ± 11.29
**LDH**	125	673.20 ± 317.47	49	349.67 ± 96.31
**WBC (Per 1000)**	125	11.98 ± 8.84	49	9.45 ± 13.35
**RBC (Per million)**	125	5.56 ± 0.82	49	5.28 ± 0.59
**HGB**	125	13.25 ± 3.16	49	13.18 ± 2.10
**HCT**	125	40.58 ± 10.14	49	39.85 ± 7.23
**PLT**	125	355.80 ± 233.87	49	524.16 ± 87.62
**Neutrophil (Per 1000)**	125	8.40 ± 3.77	49	5.01 ± 1.37
**Lymphocyte (Per 1000)**	125	1.77 ± 1.73	49	1.95 ± 0.51

RSBI: rapid shallow breathing index

Pao2/Fio2 ratio: Pressure of Arterial Oxygen to Fractional Inspired Oxygen Concentration

FIO2: Fractional Inspired Oxygen Concentration

NLR: neutrophil/lymphocyte ratio

CRP: C-reactive protein

LDH: Lactate dehydrogenase

WBC: White blood cell

RBC: Red blood cell

HGB: Hemoglobin

HCT: Hematocrit

PLT: Platelet

The results of Spearman’s correlation coefficient test show that baseline cystatin C level has a direct and highly significant linear relationship with baseline creatinine level of patients (*P < 0.001*; r: 0.926). ). The relationship between baseline cystatin C level and baseline level of other inflammatory factors was not significant, Table 3.

**Table 3:** Correlation of Cystatin C and baseline values of Biochemical and inflammatory factors^*^

Biochemical and Inflammatory factors	Correlation coefficient N = 125	P-value
**Creatinine**	0.926	**< 0.001** ^**^
**NLR**	-0.085	0.347
**Ferritin**	0.142	0.113
**CRP**	0.175	0.051
**LDH**	0.167	0.063
**Neutrophil**	-0.039	0.667
**Lymphocyte**	0.084	0.351

*Spearman was used for analyzing bivariate correlation;** Correlation is significant at the 0.01 level

NLR: neutrophil/lymphocyte ratio

CRP: C-reactive protein

LDH: Lactate dehydrogenase

Lymphopenia was reported in 41.6% (n = 52). The mean baseline cystatin C level in patients with lymphopenia was 1.20 ± 0.45 mg/L and had no significant difference from other patients (*P = 0.182*). The baseline level of other inflammatory factors, including creatinine, ferritin, NLR, LDH, and CRP, according to lymphopenia, is shown in Table 4. As indicated, the average level of ferritin, NLR, and CRP in patients with lymphopenia was significantly higher than in other patients.To identify possible confounding variables, the demographic and clinical characteristics of patients with and without lymphopenia are compared in Table 4. The mean age of patients with lymphopenia was 63.50 ± 16.92 years which was about 20 years higher than the average age (43.69 ± 16.94) in people who did not have lymphopenia (*p < 0.001*).

**Table 4:** Baseline level of Biochemical and inflammatory factors and Demographic and clinical characteristics of patients according to lymphopenia

Biochemical and Inflammatory factors	Lymphopenia-Positive N = 52		Lymphopenia-Negative N = 73			P-value	
**Cystatin C**	1.20 ± 0.45		1.58 ± 1.13			0.182	
**Creatinine**	1.19 ± 0.45		1.51 ± 1.04			0.216	
**Ferritin (Per 100)**	7.78 ± 4.42		5.16 ± 3.49			**0.001**	
**NLR**	9.72 ± 5.03		3.64 ± 1.20			**< 0.001**	
**LDH (Per 100)**	7.27 ± 3.98		6.34 ± 2.39			0.468	
**CRP**	108.75 ± 66.55		76.69 ± 45.64			**0.002**	
**Demographic**							
**Age**		63.50 ± 16.92		43.69 ± 16.94	< 0.001^1^		
**Sex; n (%)**							
**Male**		28(54)		43(60)	0.574^2^		
**Female**		24(46)		30(40)			
**Positive report for smoking**		14(27)		29(40)	0.137^2^		
**BMI**		27.95 ± 5.51		25.19 ± 5.11	0.007^1^		
**Positive report for comorbidities**							
**Diabetic**		17(33)		14(19)	0.085^2^		
**Hypertension**		18(35)		16(22)	0.116^2^		
**Dyslipidemia**		18(35)		16(22)	0.116^2^		
**Cardiovascular disease**		20(38.50)		9(12)	0.001^2^		
**Asthma**		4(8)		19(26)	0.009^2^		
COPD^*^		16(31)		23(31.50)	0.930^2^		
CKD^**^		5(10)		13(18)	0.198^2^		
**Liver diseases**		6(11.50)		7(10)	0.725^2^		

*Chronic obstructive pulmonary disease; ** Chronic kidney disease; (1) Mann-Whitney test was used for comparison; (2) Chi-square test was used for comparison

NLR: neutrophil/lymphocyte ratio

CRP: C-reactive protein

LDH: Lactate dehydrogenase

Moreover, patients with lymphopenia were overweight (mean of BMI = 27.95 ± 5.51 kg/m^2^), while other patients were almost not overweight (25.19 ± 5.11 kg/m^2^) (*p = 0.007*). Most patients in both groups were men (*P = 0.574*). Comparing the distribution of comorbidities in two groups, a significant difference was observed in cardiovascular disease and asthma. Several patients with cardiovascular disease were reported more in the group with lymphopenia, while this number was lower in the case of asthma. On the other hand, considering that no significant relationship was observed between the demographic and clinical factors of the patients with ferritin, NLR, and CRP, it was not necessary to repeat the analysis to control the confounding variables.

The average score of the severity of lung involvement was 31.42 ± 10.80. Also, there is a direct and highly significant linear relationship between baseline cystatin C level and lung involvement severity score (r = 0.890, *P < 0.001*). The correlation between the severity of lung involvement and the baseline values of other inflammatory factors, including creatinine, ferritin, NLR, LDH, and CRP, are shown in Table 5. It was significant only in the case of creatinine (r = 0.851, *p < 0.001*).

**Table 5:** Correlation of basic values of inflammatory factors and lung involvement severity score^*^

Biochemical and Inflammatory factors	Correlation coefficient N = 125	P-value
**Cystatin C**	0.890	**< 0.001** ^**^
**Creatinine**	0.851	**< 0.001** ^**^
**Ferritin**	0.114	0.205
**NLR**	-0.041	0.654
**LDH**	0.149	0.097
**CRP**	0.105	0.244

*Spearman was used for analyzing bivariate correlation;** Correlation is significant at the 0.01 level

NLR: neutrophil/lymphocyte ratio

CRP: C-reactive protein

LDH: Lactate dehydrogenase

The simultaneous effect of baseline cystatin C and creatinine values on the severity of lung involvement were evaluated using a generalized linear model. Results showed that baseline cystatin C has a higher diagnostic power in predicting the severity of lung involvement (B = 3.88 ± 1.74, *p = 0.026*). So, for each one unit increase in the baseline values of cystatin C and creatinine, the average score of the severity of lung involvement will increase by 14 and 11 units, respectively (*p < 0.001* for both).

Out of 125 patients, 53% (n = 66) were intubated and 47% (n = 59) were not intubated. The mean baseline cystatin C level in intubated patients was 1.48 ± 1.09 mg/L and in non-intubated patients was 1.35 ± 0.69 mg/L. And in this regard, there was no significant difference between the two groups of patients (*P = 0.899*).

The analysis of the basic values of other inflammatory factors, including creatinine, ferritin, NLR, LDH, and CRP, according to the intubation status of the patients, is shown in Table 6. As can be seen, the average level of ferritin, LDH, and CRP in intubated patients was significantly higher than in other patients.To identify possible confounding variables, the demographic and clinical characteristics of patients with and without intubation are compared in Table 6. The mean age of intubated patients was about 8 years, and the mean BMI was about 3 units higher than non-intubated patients (*P-value* equal to *0.017* and *< 0.001*, respectively). Comparing the comorbidities distribution in these two groups of patients was significant in terms of dyslipidemia and cardiovascular disease. So, the number of patients with dyslipidemia and cardiovascular disease in intubated patients was 38% and 33%, respectively, and in non-intubated patients, 15% and 12%, respectively (*P-value* in both comparisons equal to *0.005*).

**Table 6:** Baseline level of biochemical and inflammatory factors and Demographic and clinical characteristics according to intubation*

Biochemical and Inflammatory factors	With intubation N = 66			Without intubation N = 59		P-value
**Cystatin C**	1.48 ± 1.09			1.35 ± 0.69		0.899
**Creatinine**	1.40 ± 0.94			1.35 ± 0.75		0.813
**Ferritin (Per 100)**	7.81 ± 3.65			4.51 ± 3.89		**< 0.001**
**NLR**	6.81 ± 4.88			5.46 ± 3.96		0.755
**LDH (Per 100)**	7.83 ± 3.19			5.49 ± 2.68		**< 0.001**
**CRP**	114.95 ± 59.62			62 ± 39		**< 0.001**
**Demographic**						
**Age**		55.86 ± 17.42	47.54 ± 20.88		**0.017** ^**1**^	
**Sex; n (%)**						
**Male**		35(53)	36(61)		0.368^2^	
**Female**		31(47)	23(39)			
**Positive report for smoking**		23(35)	20(34)		0.911^2^	
**BMI**		27.86 ± 5.85	24.64 ± 4.38		**< 0.001** ^**1**^	
**Positive report for comorbidities**						
**Diabetic**		18(27)	13(22)		0.498^2^	
**Hypertension**		20(30)	14(24)		0.410^2^	
**Dyslipidemia**		25(38)	9(15)		**0.005** ^**2**^	
**Cardiovascular disease**		22(33)	7(12)		**0.005** ^**2**^	
**Asthma**		11(17)	12(20)		0.597^2^	
COPD^*^		20(30)	19(32)		0.819^2^	
CKD^**^		12(18)	6(10)		0.203^2^	
**Liver diseases**		8(12)	5(8.50)		0.505^2^	

*Chronic obstructive pulmonary disease; ** Chronic kidney disease; (1) Mann-Whitney test was used for comparison; (2) Chi-square test was used for comparison

NLR: neutrophil/lymphocyte ratio

CRP: C-reactive protein

LDH: Lactate dehydrogenase

On the other hand, the relationship between demographic and clinical factors of patients with ferritin, NLR, and CRP was significant only for CRP and dyslipidemia (*P = 0.020*). So the mean CRP in patients with dyslipidemia was 102.85 ± 50.50 mg/dl; in other patients, it was 85.14 ± 59.14.

Based on this, using logistic regression, the relationship between CRP and intubation status was still significant by adjusting the patients’ dyslipidemia status as a confounding variable. By keeping other conditions constant, for each unit increase in CRP, the chance of intubation increased by 1.02 times (*P < 0.001*). Moreover, keeping other conditions constant, the chance of intubation in patients with dyslipidemia was significantly 2.82 times that of patients without dyslipidemia (*P = 0.033*).

The average ventilation time of intubated patients was 2.72 days, and the relationship between baseline cystatin C level and the average ventilation time of intubated patients was insignificant (r=-0.162, *P = 0.195*). However, the average ventilation time of intubated patients showed a direct and incomplete linear relationship with the baseline values of NLR and CRP (*P-value* equal to *0.012* and *0.024*, respectively), Table 7.

**Table 7:** Correlation of basic values of inflammatory factors and ventilation time^*^

Biochemical and Inflammatory factors	Correlation coefficient N = 66	P-value
**Cystatin C**	-0.162	0.195
**Creatinine**	-0.140	0.263
**Ferritin**	0.139	0.265
**NLR**	0.308	**0.012** ^**^
**LDH**	0.165	0.186
**CRP**	0.278	**0.024** ^**^

*Spearman was used for analyzing bivariate correlation;** Correlation is significant at the 0.05 level

NLR: neutrophil/lymphocyte ratio

CRP: C-reactive protein

LDH: Lactate dehydrogenase

The acute kidney injury (AKI) report was positive in 11% of patients (n = 14). The mean baseline cystatin C level in patients with AKI was 2.41 ± 1.43 mg/L and significantly higher than patients without AKI (*P > 0.001*).

Baseline values of other inflammatory factors, including creatinine, ferritin, NLR, LDH, and CRP, according to AKI status, are shown in Table 8. Among the above factors, the average baseline creatinine level in patients with AKI was significantly higher by 1.27 units than in other patients (*P < 0.001*).

**Table 8:** Baseline level of biochemical and inflammatory factors according to AKI status*

Biochemical and Inflammatory factors	AKI-Positive N = 14	AKI-Negative N = 111	P-value
**Cystatin C**	2.41 ± 1.43	1.30 ± 0.76	**< 0.001**
**Creatinine**	2.50 ± 1.60	1.23 ± 0.58	**< 0.001**
**Ferritin (Per 100)**	5.64 ± 3.91	6.33 ± 4.13	0.650
**NLR**	5.36 ± 4.58	6.27 ± 4.50	0.093
**LDH (Per 100)**	6.35 ± 2.97	6.78 ± 3.20	0.793
**CRP**	82.07 ± 31.59	90.95 ± 59.75	0.934

* Mann-Whitney test was used for comparison

NLR: neutrophil/lymphocyte ratio

CRP: C-reactive protein

LDH: Lactate dehydrogenase

The average hospitalization duration of the patients was 10.70 ± 5.74 days, and there was no significant relationship between the baseline cystatin C level of the patients and hospitalization duration (r=-0.049, *P = 0.586*). A direct and incomplete linear relationship was observed between ferritin (r = 0.219, *P = 0.014*) and CRP (r = 0.241, *P = 0.007*) with hospitalization duration.

34.4% (n = 43) of patients expired in the hospital, and the mean baseline cystatin C level of this group of patients was 1.58 ± 0.90 mg/L which was significantly higher than other patients (1.35 ± 0.94 mg/L, *P = 0.002*). Moreover the mean baseline values of creatinine (1.32 ± 0.89 mg/L vs. 1.49 ± 0.78 mg/L, *p = 0.012*), ferritin (4.98 ± 3.45 mg/L vs. 8.69 ± 4.19 mg/L, *p < 0.001*), LDH (5.91 ± 2.49 mg/L vs. 8.29 ± 3.73 mg/L, *p < 0.001*) and CRP (76.54 ± 57.18 mg/L vs. 115.53 ± 48.55 mg/L, *p < 0.001*) in patients who died in the hospital was significantly higher than other patients.

Backward logistic regression analysis showed that the simultaneous effect of baseline values of cystatin C, creatinine, ferritin, LDH, and CRP on the occurrence of death in the hospital only remained significant for ferritin and LDH; So that for each unit of increase in baseline values of ferritin and LDH, the chance of dying in the hospital increased one-fold (*P-value < 0.001* and *0.010* respectively), Table 9.

**Table 9:** Effect of baseline biochemical and inflammatory factors on in-hospital death^*^

Biochemical and Inflammatory factors	OR (95%CI) N = 125	P-value
Cystatin C	1.22 (0.80, 1.88)	0.347
Creatinine	1.10 (0.27, 4.40)	0.892
Ferritin	1.002 (1.001, 1.003)	**< 0.001**
LDH	1.002 (1, 1.004)	**0.010**
CRP	1.003 (0.99, 1.01)	0.555

* Backward logistic regression was used for comparison

CRP: C-reactive protein

LDH: Lactate dehydrogenase

To identify possible confounding variables, the demographic and clinical characteristics of patients who died in the hospital are compared with other patients. Considering that there was no significant difference between the two groups of patients in terms of demographic and clinical characteristics, it was not necessary to repeat the analysis in an adapted manner to check the relationship between the baseline values of cystatin C and other inflammatory factors.

Among the 82 patients discharged from the hospital, 9.75% (n = 8) expired within one month after discharge. The average baseline cystatin C level of this group of patients was 1.58 ± 1.68 mg/L, which was not significantly different from the level of patients who survived during this period (1.41 ± 0.86 mg/L, *P = 0.593*). Comparing the mean values of other inflammatory factors in patients who died within one month after discharge with those who survived showed that it was significant only in ferritin (*P = 0.004*). The mean baseline ferritin level in patients who died within one month after discharge was about 4 units higher than in patients who survived during this period (8.38 ± 2.45 mg/L vs. 4.61 ± 3.35 mg/L, respectively).

Among the 74 patients who survived one month after discharge from the hospital, 4 patients (5%) died within 3 months after discharge. The average baseline cystatin C level of this group of patients was 1.00 ± 0.021 mg/L was not significantly different from the patients who survived during this period (1.44 ± 0.94 mg/L, *P = 0.510*). Other inflammatory factors were not significantly different between the two groups.

In the investigation of the relationship between baseline values ​​of cystatin C and other inflammatory factors with death within one month and 3 months after discharge, there was not enough sample in the subgroup of expired patients to compare with alive patients. Therefore the observed relationship is subject to random error.

The relationship between cystatin C level and baseline respiratory factors of patients showed that there is only a direct and incomplete linear relationship between baseline breathing rate and cystatin C level (r = 0.202, *P = 0.024*).

Generalized linear models (GLM) showed that for each unit increase in the baseline breathing rate, the breathing rate of the patients increased by 0.23 units in the follow-up measurement.

Results showed no correlation between baseline cystatin C level and follow-up respiratory factors, Table 10.

**Table 10:** Correlation of follow-up values of respiratory indexes and Cystatin C^*^

Respiratory Factors	Correlation Coefficient N = 49	P-value
**RSBI**	-0.235	0.104
**Pao2/Fio2 ratio**	0.223	0.124
**FIO2**	-0.054	0.714
**Oxygen saturation**	0.109	0.456

*Spearman was used for analyzing bivariate correlation

RSBI: rapid shallow breathing index

Pao2/Fio2 ratio: Pressure of Arterial Oxygen to Fractional Inspired Oxygen Concentration

FIO2: Fractional Inspired Oxygen Concentration

## Discussion

The present cross-sectional study revealed several factors related to COVID-19 consequences. Among factors, cystatin C had a direct and highly significant linear relationship with lung involvement severity score. Besides cystatin C, creatinine is also significantly associated with lung involvement severity score. However, compared with creatinine, cystatin C had a higher diagnostic power to predict the severity of lung involvement. So for each one unit increase in the baseline values of cystatin C and creatinine, the average score of the severity of lung involvement will increase by 14 and 11 units, respectively. A recent study by Chen et al. [22] investigated the value of cystatin C for predicting the prognosis of COVID-19 patients. Their study showed that increases in cystatin C were associated with long-term lung damage. In confirming the results of a study done by Chen and our study, another study also showed similar results in COVID-19 patients. A total of 273 COVID-19 patients enrolled with imaging progression in the Yang study. The result showed that parameters including homocysteine, urea, creatinine, and serum cystatin C were significantly higher in imaging progression patients [23].

Among 125 patients who participated in this study, 53% were intubated, and the present study showed that ferritin, CRP, and LDH as inflammatory factors were significantly higher in intubated patients than in others. Recently, a published study investigated the difference in the basic factors and consequences of COVID-19 according to the time of intubation. This study divided patients into “early intubation” and “late intubation”. Early intubated patients (defined as intubation between 4 and 24 h after admission to the hospital) had a lower ferritin level than late intubation patients (intubation between 5 and 10 days after admission). At the same time, CRP was higher in that group of patients [24].

In the present study, body mass index (BMI) was about 3 units higher in intubated patients than non-intubated patients. The data from another research [25] showed that patients with higher BMI have more respiratory distress on hospital admission (increased dyspnea, need for supplemental oxygen, and higher respiratory rate) and higher intubation rates. These data confirm the hypothesis that a greater degree of respiratory compromise in patients with COVID-19 is related to obesity.

Although obesity and metabolic syndrome have been related to Immune system disorders and a higher risk of pneumonia [26, 27], the impact of obesity on COVID-19 severity has not yet been fully determined. Impaired respiratory mechanics, restrictive lung physiology caused by excess body weight, and poor pulmonary reserve may play a role in the effect of obesity on severe respiratory distress in patients with COVID-19 [26–29].

Results of our study showed that the average ventilation time of intubated patients has a direct and incomplete linear relationship with the baseline level of NLR and CRP. Several studies [30, 31] that investigated prediction risk scores for ventilation in patients with COVID-19 revealed that CRP could predict the need for ventilation in these patients. These results were in line with the results of the present study. Moreover, other studies [9, 32, 33] illustrated that NLR is a good predictor of mechanical ventilation in patients with COVID-19. Another study in China indicated that high NLR > 3.13 in a company with a high cytokine response could be a good indicator of the exacerbating clinical condition of patients with COVID-19 [34].

Many studies have been conducted on the predictive power of cystatin C and creatinine for the occurrence of AKI. One of these studies is a meta-analysis that analyzed the results of 19 studies [35]. The diagnostic odds ratio (OR) for serum cystatin C level to predict AKI was 23.5. Results of that study declare that serum cystatin C is a good biomarker in predicting AKI. In the present study, we found that cystatin C and creatinine were significantly higher in patients with AKI compared to other patients. Still, none of the two mentioned factors significantly predicted the chance of AKI occurrence. It should be noted that the significant result observed between baseline values ​​of cystatin C and creatinine with the occurrence of AKI in patients can be caused by random error due to the very small sample size in the subgroup of patients with AKI. For this reason, the distribution of demographic and clinical characteristics to identify potential confounding variables according to AKI status was not sufficiently powerful in the current study. Therefore, it is necessary to investigate the relationship observed in this study in future studies with a sufficient sample size and by controlling possible confounders.

The predictive value of cystatin C in the prognosis of patients with COVID-19 is rarely reported, but some studies have confirmed that cystatin C can predict the risk of death in patients with COVID-19 [5, 36–38]. In our study, 34% of patients with COVID-19 expired in the hospital, and the baseline cystatin C level of these patients was significantly higher than alive patients. In addition to cystatin C, a baseline level of creatinine, CRP, ferritin, and LDH is higher in expired patients than in others. The Xiang study showed that patients with severe COVID-19 had a high level of biochemical indicators of renal function like BUN, cystatin C, and creatinine, indicating kidney damage [39]. Besides Xiang’s study, other studies have also shown the occurrence of kidney disorders in COVID-19 disease [36, 40, 41]. The incidence of AKI in patients with COVID-19 has been reported from 0.9 to 29% [9, 13, 42]. An acute proximal renal tubular injury characterized by loss of brush margin, vacuolar degeneration, dilation of the tubular lumen with cell fragments, necrosis, and epithelial exfoliation has been seen in patients with COVID-19 [41]. The total mortality rate of patients with renal involvement was significantly higher than that of other patients without renal involvement (11.2% vs. 1.2%, respectively) (42), proposing that the mortality rate of COVID-19 may be related to renal involvement.

## Conclusion

In conclusion, cystatin C and other inflammatory factors such as ferritin, LDH and CRP can help the physician predict the consequences of COVID-19. Timely diagnosis of these factors can help reduce the complications of COVID-19 and better treat this disease. More studies on the consequences of COVID-19 and knowing the related factors will help treat the disease as well as possible.

## Data Availability

The datasets used during the current study are available from the corresponding author on reasonable request.
